# Advanced imaging of fetal cardiac function

**DOI:** 10.3389/fcvm.2023.1206138

**Published:** 2023-05-23

**Authors:** Henriette Kühle, Steven K. S. Cho, Nathaniel Barber, Datta Singh Goolaub, Jack R. T. Darby, Janna L. Morrison, Christoph Haller, Liqun Sun, Mike Seed

**Affiliations:** ^1^ Division of Cardiology, Department of Pediatrics, The Hospital for Sick Children, University of Toronto, Toronto, ON, Canada; ^2^ Department of Cardiac and Thoracic Surgery, University Hospital Magdeburg, Otto von Guericke University Magdeburg, Magdeburg, Germany; ^3^ Division of Cardiac Surgery, Department of Pediatrics, The Hospital for Sick Children, University of Toronto, Toronto, ON, Canada; ^4^ Department of Physiology, Faculty of Medicine, University of Toronto, Toronto, ON, Canada; ^5^ Early Origins of Adult Health Research Group, University of South Australia, Adelaide, SA, Australia; ^6^ Translational Medicine Program, The Hospital for Sick Children, University of Toronto, Toronto, ON, Canada; ^7^ Research Institute, The Hospital for Sick Children, University of Toronto, Toronto, ON, Canada; ^8^ Department of Diagnostic Imaging, The Hospital for Sick Children, University of Toronto, Toronto, ON, Canada

**Keywords:** fetal cardiac function, echocardiography, magnetic resonance imaging, speckle tracking, strain

## Abstract

Over recent decades, a variety of advanced imaging techniques for assessing cardiovascular physiology and cardiac function in adults and children have been applied in the fetus. In many cases, technical development has been required to allow feasibility in the fetus, while an appreciation of the unique physiology of the fetal circulation is required for proper interpretation of the findings. This review will focus on recent advances in fetal echocardiography and cardiovascular magnetic resonance (CMR), providing examples of their application in research and clinical settings. We will also consider future directions for these technologies, including their ongoing technical development and potential clinical value.

## Introduction

As we increasingly regard the unborn fetus as a patient and develop improved prenatal diagnosis and treatment of a range of fetal conditions, we require more sophisticated approaches to assessing fetal wellbeing. There is no better example of the evolution of prenatal diagnosis and therapy than our imaging approach to the fetal heart and cardiovascular system. The prenatal diagnosis of congenital heart disease has transformed the perinatal management of these common birth defects, resulting in improved outcomes and offering families an opportunity to make informed choices about a range of management options. The transplacental treatment of fetal arrhythmias represents one of the earliest and most widely used forms of fetal therapy. With the advent of minimally invasive catheter interventions for severe forms of congenital heart disease, we have learned that in selected cases it may be possible to modify the natural history of congenital heart disease, thereby avoiding the need for single ventricle palliation while improving fetal development. The advent of increasingly sophisticated approaches to fetal cardiac imaging has played an essential role in this story, with advances in the assessment of fetal cardiac function occurring in tandem with new treatments (1, 2). For example, abnormalities of myocardial loading and performance manifesting as cardiac enlargement, reduced contractility or hydrops may be observed in the setting of congenital heart lesions such as Ebstein's anomaly or critical aortic stenosis as well as other fetal cardiac conditions such as cardiomyopathies and fetal arrhythmias (3–5). The severity of the cardiac compromise in these settings may determine the indication for fetal treatment and serve as a marker of response to therapy. Similarly, the detection of subtle abnormalities of cardiovascular physiology or heart function may provide clues to the presence of extra-cardiac pathologies, including fetal growth restriction (FGR), fetal anemia and twin-to-twin-transfusion syndrome (TTTS).

Echocardiography is a widely available and non-invasive technique that is used to identify structural heart disease and predict prognosis (6, 7). Through technical advances and growing expertise, fetal echocardiography, which was first described in 1964, is now increasingly integrated into routine obstetric ultrasound (8–10). Ultrasound provides excellent contrast between the myocardium and blood pool with high spatial and temporal resolution, allowing exquisite visualization of cardiac anatomy and function. Conventional 2-dimensional (2D) echocardiography is used to visualize myocardial deformation and measure fractional shortening, obtained with standard grey-scale imaging or M-mode and converted to ejection fraction using the cube method or Teichholz formula. By contouring the endocardial border in systole and diastole from 2D echo images, more accurate measures of ejection fraction can be obtained using Simpson's method. Ventricular function can also be further quantified *in utero* using M-mode echocardiography. A variety of Doppler techniques further enhance the assessment of cardiac physiology, as well as providing information about vessel tone and resistance in the various fetal vascular beds. Color Doppler allows direct visualization of the flow of blood through the heart and vessels, while pulsed Doppler provides peak and mean velocities across valves, vessels, and cardiac chambers, as well as the characterization of flow patterns throughout the cardiac cycle. Thus, left ventricular stroke volume can be calculated as the product of left ventricular outflow tract area and mean flow velocity. Systemic and pulmonary venous flow patterns and inflow velocity profiles across the atrioventricular valves provide information about diastolic function. Systolic and diastolic function can be further interrogated using Tissue Doppler Imaging (TDI), while three-dimensional (3D) or four-dimensional (4D) echocardiographic techniques yield alternative approaches to measuring chamber volumes, stroke volume and ejection fraction. Speckle tracking provides an approach to regional and global strain and strain rate imaging. However, accurate prenatal diagnosis by fetal echocardiography depends on the skill and experience of the operator, particularly in the setting of complex cardiac anatomy. The correct interpretation of the imaging may be challenging when ultrasound imaging is hampered by poor visualization of the fetal heart resulting from adverse maternal body habitus, fetal positioning or oligohydramnios. With advancing gestation, image quality may also be affected by the progressive calcification of the fetal ribs and spine (11) and by the increasing distance between the ultrasound probe and the fetal heart (12). At younger gestations, vigorous fetal motion may compromise fetal cardiac imaging, while difficulties in obtaining certain cardiac views may be further aggravated by the more horizontal orientation of the long heart axis due to a relatively large fetal liver (13, 14). The fetal electrocardiogram (ECG) is not readily available for advanced techniques that require synchronization of image collection to the cardiac cycle, which has led to the implementation of alternative gating methods i.e., via anatomic M-mode, or mitral movement (12, 15–17).

The application of fetal Cardiovascular Magnetic Resonance has been slower than other fetal Magnetic Resonance Imaging (MRI) techniques, which may partly reflect the challenges associated with obtaining diagnostic cardiac imaging of appropriate quality in the fetus with MRI. Cardiac MRI provides accurate measurements of right and left ventricular end-diastolic and end-systolic volumes, and therefore stroke volume and ejection fraction, typically through the segmentation of a stack of short axis cine images through the ventricular mass. Thus, fetal CMR typically generates thicker imaging planes than ultrasonographic techniques (6), and usually requires significant attention to postprocessing, but can result in improved image quality when ultrasound imaging is hampered by maternal obesity, oligohydramnios, or rib calcification, especially in the later stages of pregnancy (1, 18, 19). Fetal CMR is challenged by the lack of an ECG signal for cardiac gating, the high fetal heart rate and, most importantly, by frequent fetal body motion. Thus, a series of technical adaptations have been required to make fetal CMR feasible, including several alternative gating methods including self-gating (20–25), retrospective metric optimized gating (26–29), and CTG- or Doppler ultrasound cardiac gating (30–39) and accelerated acquisition strategies employing under-sampling methods and motion correction. As a result of the innate tradeoff between scan time and signal-to-noise ratio (SNR), fetal CMR has significantly lower spatial and temporal resolution than ultrasound. Cine phase contrast MRI provides information about ventricular function by providing accurate measurements of vessel flow. Myocardial tagging and feature tracking are MRI techniques for measuring strain and strain rate. A sensitive marker of LV function that is available by MRI is LV torsion, whereby apical and basal segments rotate in opposite directions. Other non-invasive techniques for assessing ventricular function such as nuclear medicine and computed tomography typically require ionizing radiation and the injection of contrast agents, making them unsuitable for fetal imaging. Contraindications such as claustrophobia can limit the application of fetal CMR, which may also be poorly tolerated due to the requirement for immobilization. This issue is particularly relevant in the late gestation (40, 41). However, fetal CMR is gaining in popularity as an adjunct to fetal echocardiography and has been recommended by the American Heart Association in the setting of complex viscero-sital cardiac abnormalities and for its evolving role in assessing fetal cardiovascular physiology (1, 34, 37, 41–43). Some traditional strengths of CMR in postnatal heart disease include the delineation of vascular anatomy by angiography, as well as accurate ventricular volumetry for assessing cardiac chamber sizes and function, and quantifying vessel flow and valvar regurgitation and myocardial tissue characterization. While administering gadolinium contrast agents is contraindicated in pregnant patients, a combination of late gadolinium enhancement and cardiac cine imaging has been used to evaluate the biology of myocardial infarctions and ventricular function in preclinical fetal and postnatal sheep studies (44, 45). A combination of vessel flow and magnetic resonance oximetry has also been applied in sheep to assess the distribution of blood flow and oxygen transport across the fetal circulation, and as an approach to comprehensively quantifying the placental oxygen transfer (31, 46–51). Fetal CMR has been used in human pregnancies to study the effect of congenital cardiac malformations on fetal cardiovascular physiology, particularly through the implementation of cine phase contrast for quantifying vessel flow and relaxometry for characterizing vessel oxygen saturation and hematocrit, techniques that have also been applied in the setting of fetal growth restriction and anemia (19, 25, 29, 33, 37, 38, 50, 52–64).

For this review, authors specialized in different fields worked together to review literature on advancements in the technology to assess fetal heart function focusing on the most recent findings and referring to their first implementations and development when applicable.

## Approaches to assessing cardiac function

### Fetal echocardiography

**Figure 1:**
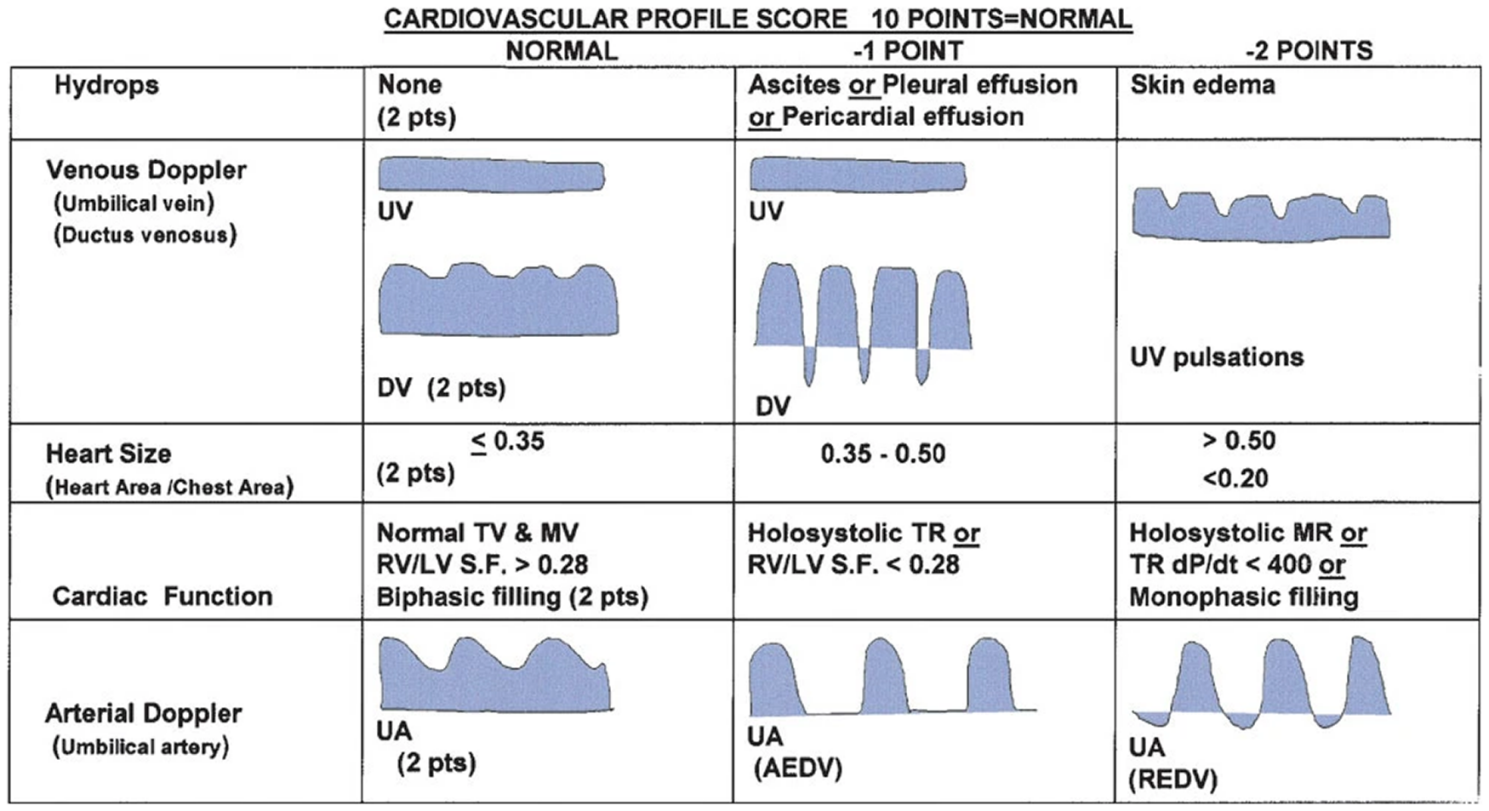
Cardiovascular profile score (CVPS) to assess fetal heart function, adapted from (89).

**Figure 2:**
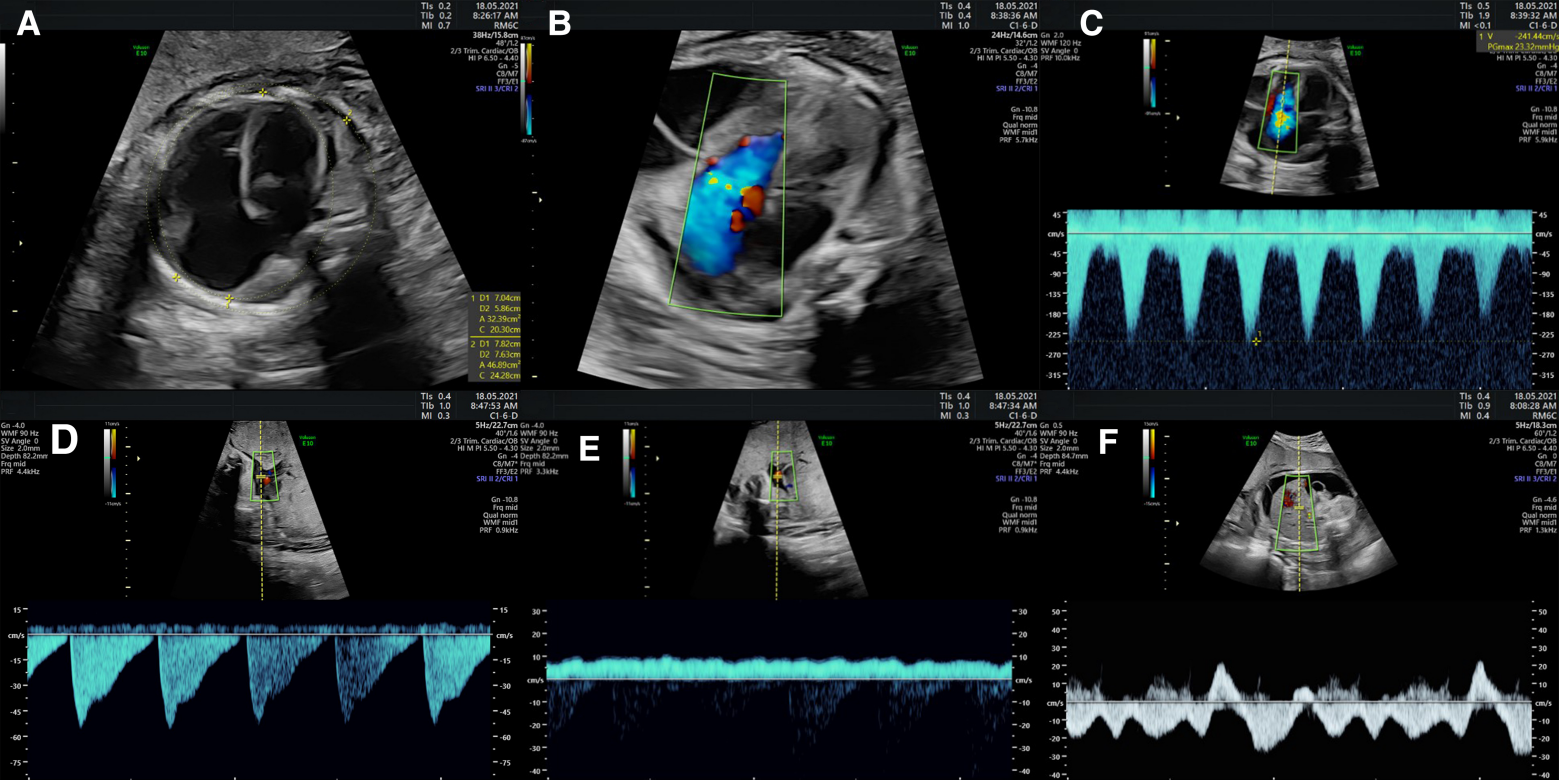
The assessment of fetal cardiac function and cardiovascular profile in Ebstein anomaly showing severe cardiomegaly (cardiothoracic ratio: 0.69) with severely dilated right atrium and right ventricle and compressed lungs and mild to moderately reduced RV function, fetal hydrops with a small pericardial effusion, significant ascites and scalp edema. The Dopplers reveal intermittent a-wave reversal in the ductus venosus and umbilical vein notching suggestive of elevated cardiac filling pressures and intermittently absent diastolic flow in the umbilical artery consistent with a systemic steal. (**A**) Four chamber view suggesting severe cardiomegaly (cardiothoracic ratio: 0.69) and dilated right heart. (**B**) Color Doppler showing tricuspid regurgitation. (**C**) Doppler of tricuspid valve showing biphasic flow with high velocity. (**D**) Intermittently absent diastolic flow in the umbilical artery. (**E**) Umbilical vein notching. (**F**) The intermittent a-wave reversal in the DV.

Several guidelines providing details regarding the correct acquisition and interpretation of fetal echocardiographic functional parameters have been published (5, 65–73). Additional resources detail a range of well-established techniques for assessing fetal cardiac function by echo (3, 5, 6, 18, 65, 74–77). Summaries of some of the more popular techniques for assessing ventricular function by fetal echo are provided below. Most quantitative fetal echocardiographic techniques for assessing myocardial function have similar limitations in the fetus as they do in the postnatal heart, which includes finite reproducibility resulting from challenges in obtaining correct alignment (78). In common with most non-invasive methods for assessing myocardial function, standard echocardiographic parameters such as ejection fraction or ventricular fractional area change are representative of the load-dependent pumping function of the ventricle, rather than cardiomyocyte contractility or changing cavity pressure in the fetal heart (79, 80). Evaluation of strain rate, on the other hand, mainly reflects maturational changes in the myocardium while being relatively independent of loading conditions (81). Scoring systems like the fetal cardiovascular profile score (CVPS), shown in Figure 1, which incorporates cardiac functional indices with other imaging and Doppler findings including extracardiac vessels, as well as more specific measures of cardiac cycle intervals such as the myocardial performance index and Tei index have been established in the fetus (3, 6, 18, 82–89). Figure 2 shows an example of the application of 2D echocardiography and Doppler to calculate the CVPS in a fetus with Ebstein's anomaly with severe displacement of the tricuspid valve with good LV function.

#### 2D echocardiography and M-mode

**Figure 3:**
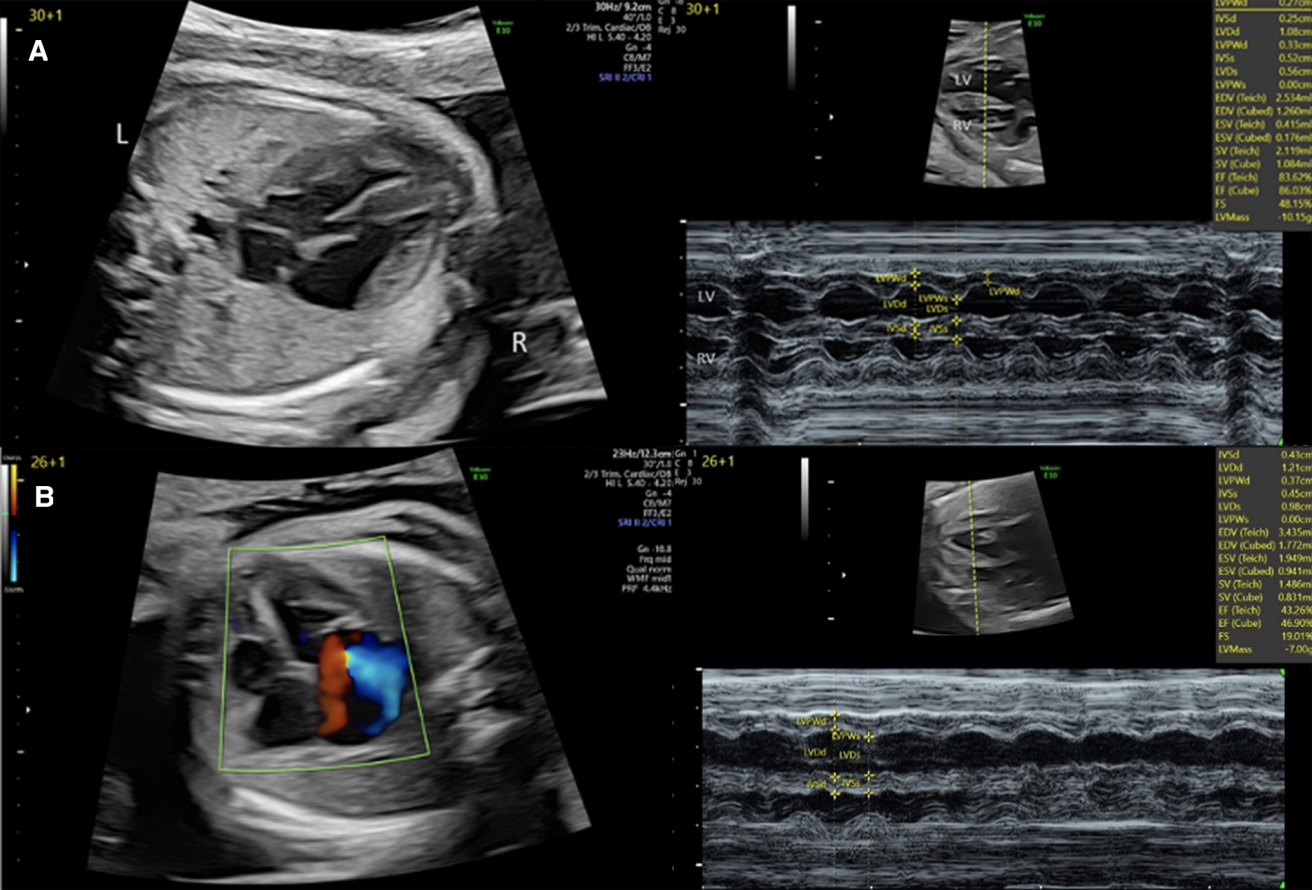
2D and M-mode assessment of left ventricular function in (**A**) a fetus with a family history of hypertrophic cardiomyopathy at 30 + 1 weeks and (**B**) a fetus with severe aortic stenosis and left ventricular endocardial fibroelastosis at 26 + 1 week.

Two-dimensional echocardiographic still-frames and cines include the standard views (abdominal situs view, four-chamber view, left ventricular outflow tract view, right ventricular outflow tract, and three-vessel view). Qualitative impairments of systolic function are typically graded as mild, moderate or severe, and frequently combined with measures of cardiothoracic ratio and other imaging findings such as vessel Dopplers, the presence or absence of hydrops and non-specific signs of fetal wellbeing such as amniotic fluid volume, and fetal growth and activity to provide an overall assessment of fetal cardiac function (3, 90). This approach, being fast and easy to execute, is frequently used as the single diagnostic method (71) despite its potential limitations. When video sequences of four-chamber view or short axis are saved, those loops can be post-processed, for example using the newer speckle tracking approach discussed below. M-mode, which captures dynamic variation in structures imaged along a single line of a 2D image, provides an approach to quantitatively analyzing systolic function, assuming the correct alignment is achieved and when standard and homogenous ventricular volumetry is present (6, 18, 68, 74, 91, 92). Stroke volume can be estimated by applying Simpson's rule, which implies an ellipsoid ventricular shape (6), while longitudinal ventricular function can be assessed based on atrioventricular plane displacement (77). Figure 3 gives an example of basic assessment of the heart by 2D echocardiography and M-Mode in a patient with a family history of hypertrophic cardiomyopathy and aortic atresia illustrating the information obtained by these techniques.

#### Doppler and tissue Doppler

Doppler techniques are routinely used in fetal medicine to measure circulatory parameters of placental function and cardiovascular physiology (93, 94). Veins continuous with the right ventricle (ductus venosus, inferior vena cava, hepatic veins) exhibit typical waveforms (6) that vary with normal maturational changes in hemodynamics occurring through gestation. By contrast, the umbilical vein flow is constant from the end of the first trimester onward (18). Altered velocity profile patterns in these veins can depict changes in fetal condition or myocardial dysfunction and serve as predictive parameters for fetal outcomes (95). Doppler is dependent on the angle of insonation and relies on the direction of blood flow being parallel to the beam for reliable measurement of velocity (6). Acquiring Doppler spectra with correct alignment can be challenging in the fetus, although mathematical angle corrections are possible (96). In fetal hypoxia, deep or reversed a-waves in the ductus venosus and pulsatile flow in the umbilical vein reflect cardiac dysfunction (64, 97–99) and correlate with poor fetal outcomes (18, 85, 100). Similarly, middle cerebral artery pulsatility is reduced due to cerebral vasodilation or “brain-sparing physiology” in the setting of fetal hypoxemia secondary to placental insufficiency or other causes (82, 93, 101–103). Pulsed Doppler is also used to assess flow patterns within the cardiac chambers and across valves (104). Atrioventricular valve flow patterns can help to evaluate diastolic function by characterizing the biphasic E and A waves, their ratio, deceleration time, isovolumetric relaxation time and change with gestation (6, 14), with normal E/A ratios usually <1 in the developing fetus (18). In congenital heart disease (CHD), abnormal and non-biphasic patterns can reflect pathologies like aortic stenosis, while reduced ratios may be seen in FGR and hydrops, although a high fetal heart rate can lead to the fusion of E and A waves (18, 81). Tricuspid inflow, hepatic vein flow, lateral tricuspid annulus TDI, and collapsibility of the inferior vena cava help to define diastolic function (71), while the ejection force in the outflow tracts reflects systolic performance (6). Stroke volume and combined ventricular output (CVO) can be calculated based on the diameters of the ventricular outflow tracts, the velocity time integral across the valve and heart rate. The outputs of the ventricles can be indexed to fetal weight using an estimate based on measurements of head size, abdominal circumference, and femur length (3). Color Doppler gives a visual overview of blood flow directions and mean velocities, which can be particularly helpful for the evaluation of cardiac symmetry and the direction and continuity of fetal shunts and valves. Color Doppler is therefore useful in the detection of congenital cardiac defects and is essential for visualizing valve regurgitation, which is an important cause of heart failure in the fetus (5, 75, 82, 105, 106). Color Doppler is also used to guide the placement of the sample volume for spectral Doppler measurements (5, 68, 107). Tissue Doppler Imaging is used to quantitatively analyse segmental wall motion and individual point changes in myocardial velocity (18). This allows the evaluation of motion- and time-related events that provide information about systolic and diastolic cardiac function (1). Valve motion of the mitral or tricuspid valve annulus relative to the relatively stable apex assesses longitudinal contractility (18, 71) typically presenting as S', E' and A' waves by pulsed wave (PW) TDI, representing systolic, early diastolic and late diastolic annular peak velocities (3, 71). However, the combination of the relatively small amount of fetal myocardial tissue and large voxel volume may result in low reproducibility, and the use of fetal TDI has mainly been limited to research settings (1). Related techniques like Color TDI and PW Doppler exhibit inconsistent results with respect to velocities in the adult heart (10%–20% higher by TDI) (78). Nevertheless, TDI relies less on image quality or border detection and has higher frame rates than 2D echocardiography or MRI (96).

#### 3D/4D echocardiography

The extension of two-dimensional imaging into real-time or reconstructed volume data sets as 3D or gated 4D sequences can be used to reproduce an unlimited number of adjustable standard 2D views. This approach to fetal cardiac imaging achieves reasonable temporal resolution combined with a relatively low acquisition time to provide a more detailed assessment of geometric and morphological changes, including stroke volume, ejection fraction and cardiac output for both ventricles (1, 6, 18, 24, 108, 109). While cheaper and more widely available than MRI (71), ultrasound 3D and 4D cardiac imaging requires dedicated transducers and expertise (1), may underestimate volumes (25, 71) and lacks temporal and spatial resolution compared with 2D imaging, especially with ungated techniques (1). Spatiotemporal image correlation (STIC) allows a volume reconstruction of a cardiac cycle by extracting temporal information from 2D images combined with other standard echocardiographic techniques and leads to a more coherent 4D reconstruction. However, this approach to cardiac cycle reconstruction requires a relatively long acquisition time that may result in motion degraded datasets (1, 5, 6). Newer iSTIC (intelligent STIC) acquisition algorithms can generate higher resolution images faster to reduce artifacts (109). Indeed, several studies have concluded that these methods are a useful addition to standard 2D images (110).

#### Speckle tracking echocardiography

Strain is defined as the grade of tissue deformation that corresponds to the change in length or thickness in response to an applied force, while strain rate is the velocity of this deformation (111). Speckles generated by ultrasound backscatter and interference form natural patterns within the myocardium, a kernel is their corresponding functional unit. The units, distance, and velocity among each other reflect strain and strain rate. Negative strain reflects the shortening that typically occurs in systole, while positive values depict diastolic lengthening (3, 12). The results are usually presented as velocity vectors within the image and as curves for strain and strain rate across the cardiac cycle. Additionally, peak values and the time and acceleration needed to reach those can be calculated. Speckle tracking and velocity vector imaging (VVI) provide an approach to semi-automatically perform post-processing of 2D four-chamber or short axis images acquired in standard examinations. This approach tracks the endocardial border of the ventricles, making automatic adjustments of the contours and providing calculations of strain, strain rate and velocity (12, 96). Concerningly, no imaging standard for this approach has yet been established (113, 114), which reflects both the differences in the software packages available and the challenge of achieving standardized imaging planes in the fetus (113–115). Examination consistency is the key to reducing variability and confounding effects (114, 116). While 2D strain and VVI use the same speckle tracking approach, the manually selected region for 2D strain usually includes the whole myocardial wall as opposed to the narrower myocardial layer tracked in VVI through algorithm border detection (111). The myocardium is usually divided into at least six segments (basal, apical, middle left and right) with possible expansion to 16- or 17-segment models for better localization of potentially restricted segments (91). Several strain types can be measured including longitudinal shortening, radial thickening, and circumferential shortening (111). In the small fetal heart, longitudinal, global strain measurements seem to be the most accurate and sensitive to pathology (14). Multiple segmental measurements do not significantly differ from global ones (96), and a globally assessed longitudinal measurement reduces the overall error by avoiding inconsistencies with the definition of exact boundaries (117). Additionally, global measurements are less affected by local noise (118), and segmental dysfunctions are less likely in the fetus compared to the adult population (111). By contrast with movement, strain and especially Lagrangian strain [strain relative to initial length, i.e., a single reference length field (12)] and its velocity (strain rate) are not dependent on adjacent regions, making them more accurate than simple velocity measurements (71, 77, 96, 119, 120). Strain measurements, influenced by extrinsic loading and intrinsic contractile force (14, 71), consider the contractility of the cardiomyocytes and changes with cardiac cavity pressure (79). Longitudinal strain and strain rate seem to reflect the fetal right ventricular dominance with strain and strain rate values between 1 and 1.5 times higher in the right compared to the left ventricle (121). Myocardial fiber orientation in the fetus with a continuous 3D meshwork in the LV contributing to systolic deformation and ventricular ejection (122) compared to the longitudinally aligned myocardium in the RV (71) limits the accuracy of reflecting longitudinal or circumferential movements in each ventricle (123). The prominent trabeculation of the RV can make border definition challenging for structures like the right atrial appendage, crista terminalis, fossa ovalis (112) or discontinued regions such as the opening of pulmonary veins into the LA and tissue elasticity can affect accuracy (14). A high contrast between the endocardium with chamber cavity and precise tracking of the walls are necessary to not underestimate strain and strain rate values (124).

**Figure 4:**
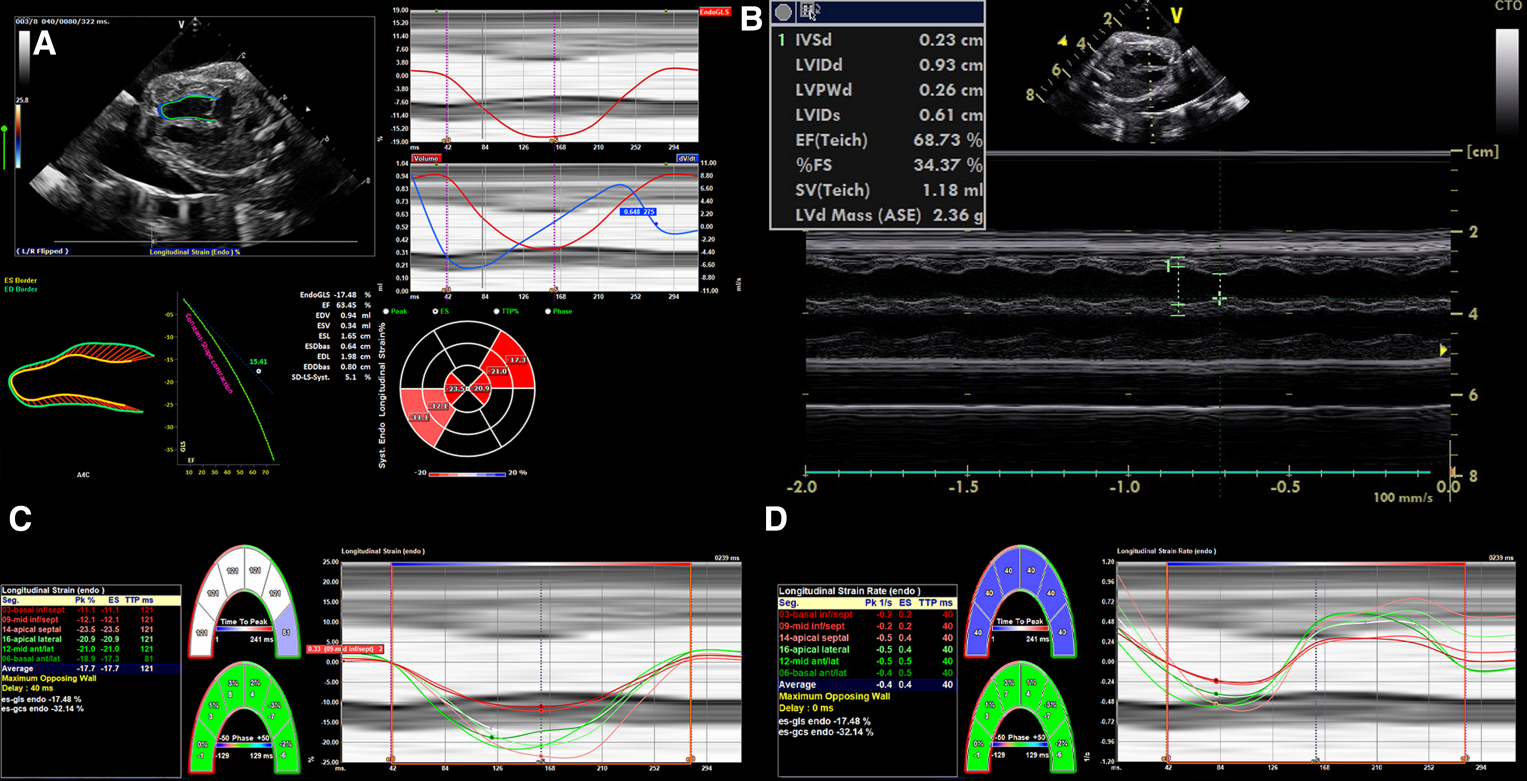
2D strain in LV and M-mode analysis from 4CV in an artificial placenta piglet model by echocardiography obtained using a voluson S6 (GE healthcare ultrasound, WI, USA) and post-processing software by TOMTEC (TOMTEC imaging systems GmbH, Germany). A gated M-Mode loop of two cardiac cycles was generated using a four-chamber cine loop, allowing for automatic LV strain analysis. Compared to the original M-mode measurements, the post-processing technique seems feasible and generated similar values for ejection fraction, with good repeatability between serial measurements. (**A**) Strain Analysis. (**B**) M-mode. (**C**) Segmental endocardial strain. (**D**) Segmental endocardial strain rate. Superimposed anatomical M-Mode behind graphs, endocardial border detection.

**Figure 5:**
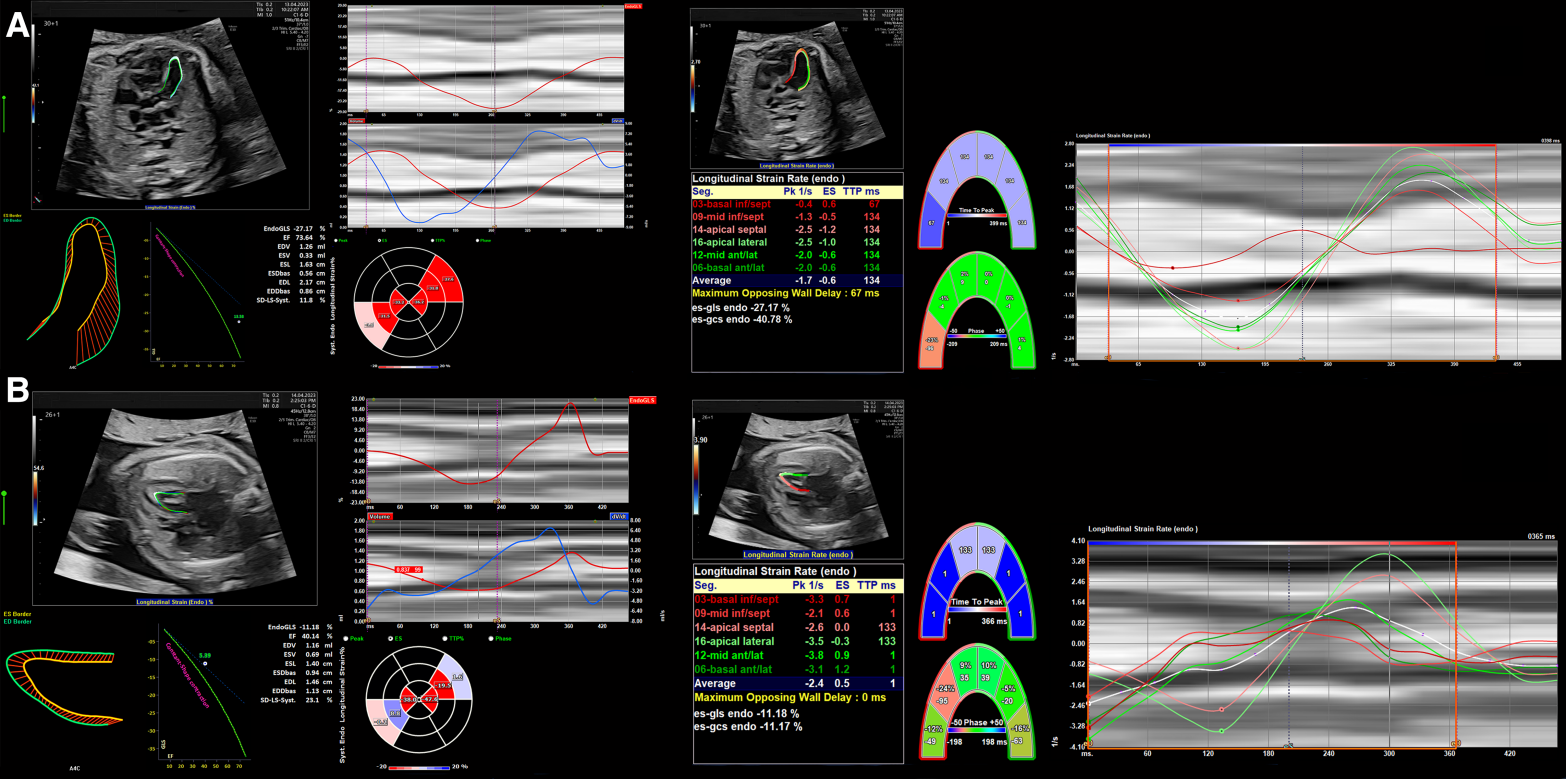
2D strain on LV analysis from 4 chamber view in (**A**) a patient with a family history of hypertrophic cardiomyopathy resulting in increased CVO and overall elevated strain measurements and (**B**) a patient with aortic stenosis resulting in reduced CVO and deviating strain measurements between the ventricular septum due to indirect movement of the hypoplastic and dysfunctional LV by the adjacent RV.

Since ECG gating is not readily available from the fetus, alternative gating techniques such as using the R-wave or M-mode to define the end-diastolic to end-systolic cycle help to keep the stored images at high reliability and frame rate (78). Beat-to-beat analysis is possible even in arrhythmia (12), and offline postprocessing is more robust and reproducible (7, 125). The large number of software packages, albeit with some variability in measurements (113, 126–128), and the availability of ultrasound machines may increase the usage of strain imaging while simultaneously decreasing comparability (122), especially since only the measurement of global longitudinal strain is available in all softwares (91, 129). Nonetheless, several studies have reported better inter- and intra-observer variability than standard techniques (7, 125, 130). Training in the acquisition and analyses of high-resolution echocardiographic images (125) is necessary to establish this method successfully (76). With more experience, 2D speckle tracking imaging may be feasible and reproducible in the fetal heart (4, 7, 113, 118). For example, strain imaging has been shown to detect functional abnormalities in human fetuses with cardiac disease (111) and hypoxemic sheep (93). Figure 4 shows cardiac strain imaging in a preclinical pig model of the artificial placenta (131, 132). In Figure 5 we applied strain analysis in the two human fetuses with CHD shown in Figure 2 and revealed changes in strain associated with reductions in combined ventricular output.

Following the first description of strain imaging for cardiac assessment by Uematsu et al. in 1995 (133) and Heimdal et al. in 1998 (134), Harada et al. were the first to report the feasibility of measuring strain in fetal hearts in 1999 (135). In postnatal subjects, Nesser et al. (136) attempted to use 3D echocardiographic imaging to generate better correlations with MRI measurements than 2D strain, which seemed to underestimate LV volumes, while Enzensberger et al. (137) showed the feasibility of 3D strain imaging. However, the small size of the fetal heart compared with adults may alter the pixel-to-myocardial value ratio, making it harder for a speckle to be tracked accurately while over-smoothing resulting from lower spatial resolution is possible (122). Furthermore, exclusion of the cardiac apex can lead to chamber foreshortening (117), resulting in an overestimation of movement between the false apex and the heart's base (92, 111). Some softwares may be unsuitable for assessing a small fetal heart, whereby the smallest available segment might already be thicker than the whole myocardial wall (7). There is currently no consensus about the optimal frame rate for strain imaging, which may be particularly relevant in the setting of high fetal heart rates. While the highest possible frame rates are recommended (i.e., above 60–100 fps) (17, 111, 123, 125, 126), accurate results have been reported with frame rates as low as 30 fps (113). Stable frame rates throughout examinations can improve comparability (114). Several studies have tracked the influence of gestational age on strain measurements with highly varying results from no correlation (13, 80, 138, 139) to decreasing values with maturation (13, 111, 117, 127, 130). This variation in findings raises the possibility that the results of fetal strain imaging are likely to be dependent on equipment and software, as well as the study population and approach, emphasizing the importance of establishing standardized techniques. In addition to healthy pregnancies, strain has been measured in fetuses that are small for gestational age, and with FGR and ventricular septal defect (81, 140). Fetal echocardiographic strain imaging has also been reported as an approach to assessing disease progression and determining the optimal timing of intervention in fetuses with maternal diabetes and pregnancies complicated by TTTS and fetal growth restriction (111).

### Fetal cardiovascular magnetic resonance imaging

Fetal MRI was initially developed for non-cardiovascular applications, which partly reflects the technical challenges associated with applying cardiovascular MRI in the fetus (150). However, in animal models these challenges can be overcome with anesthesia (151) which limits fetal body motion, and catheterization of fetal vessels, which allows for the detection of a blood pressure waveform that can be used for cardiac triggering (31, 50, 152). Thus, the cardiovascular magnetic resonance techniques that are routinely used for the non-invasive assessment of ventricular function in postnatal patients have been applied in fetal sheep to quantify myocardial mass and right and left ventricular volumes and ejection fractions. Fetal CMR with late gadolinium enhancement has also been used to detect cardiac damage in an experimental model of infarction (44, 153) and to measure vessel flow and oxygen content (48, 154) in an attempt to emulate the invasive hemodynamic measurements made in fetal sheep that have defined our modern understanding of normal fetal circulatory physiology. Similarly, through the development of alternatives to conventional ECG gating, accelerated imaging and motion correction algorithms, this combination of vessel flow and oximetry measurements has been applied in human fetuses to explore the normal circulation (154) as well as the impact of congenital heart malformations and vasoactive agents (48) on fetal circulatory physiology (37, 43). The potential role of fetal CMR as an adjunct to ultrasound has also been investigated in the setting of severe fetal hemodynamic compromise, for example in patients undergoing fetal cardiac interventions (37, 56). Fetal MRI provides information about other organ systems that may be affected by heart disease, including the lungs and brain, which can be incorporated into management planning (155). However, significant limitations of fetal CMR, including its inferior spatial and temporal resolution compared with ultrasound, its cost, and the requirement for considerable post-processing technology and time have limited the clinical implementation of fetal CMR (156). In addition, the FDA recommends limiting the use of MRI in the first trimester due to safety concerns about the impact of strong magnetic fields on embryogenesis. Accordingly, clinical trials and clinical applications of fetal CMR have typically been limited to second and third-trimester examinations (40).

#### Cine phase contrast MRI for the assessment of fetal blood flow

**Figure 6:**
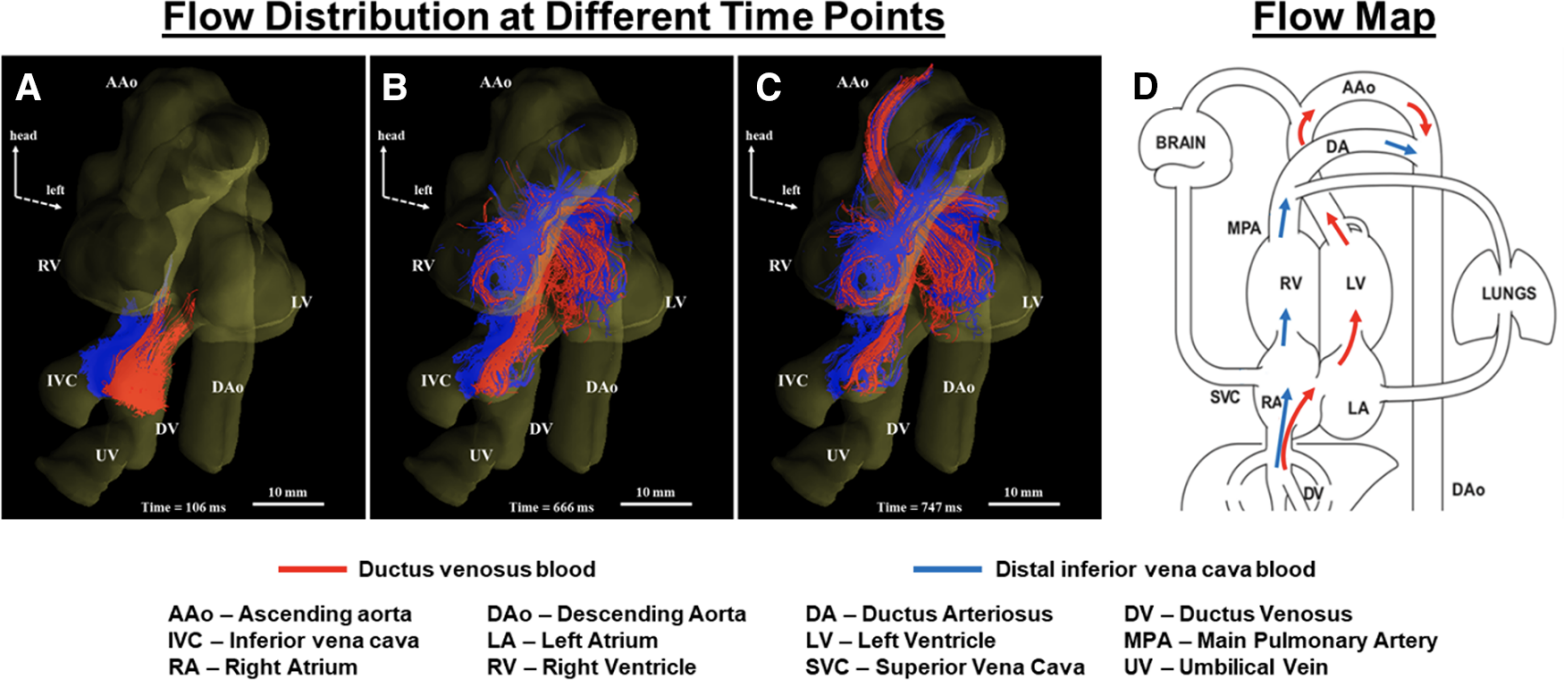
Tracing blood from inferior vena cava (IVC) and ductus venosus (DV) in a human fetal heart (**A–C)**. Coronal view showing blood from the IVC (blue) and DV (red) at different cardiac phases over two heartbeats. Streams from IVC and DV with limited mixing enter the right atrium (**A**), with blood from the DV (oxygenated) being mainly directed into the left ventricle (**B**) to supply the coronaries and upper fetal body (**C**). (**D**) Corresponding flow map. Adapted from (33).

Two-dimensional cine phase-contrast (PC) MRI is the non-invasive gold standard for vessel flow assessment in children and adults (31). However, the requirements for adequate spatial and temporal resolution limit the application of phase contrast for fetal vessel flow quantification to the larger vessels in the third trimester. The development of metric optimized gating led to the initial descriptions of fetal vessel flow quantification by CMR, and preliminary reference ranges for the distribution of the late gestation normal human fetal circulation (57). Vessel flows were also acquired using this approach in fetuses with CHD and FGR (43, 37, 58). In 2018, Goolaub et al. reported improved image quality using golden angle radial PC CMR with motion correction (35). “Four-dimensional (4D) flow” refers to the acquisition of volumetric datasets that incorporate multidirectional velocity encoding. This data can be reconstructed in any plane to measure vessel flow in an analogous approach to conventional 2D cine phase contrast imaging. 4D flow datasets can also be used to generate more complex reconstructions such as particle tracking that provide unique information about flow patterns within the heart and vessels. This approach has been applied in adult patients to assess aortic aneurysms and valvular conditions, as well as the complex hemodynamics of single ventricle physiology (157). Schrauben et al. (158) applied 4D flow in fetal sheep to visualize the streaming of the umbilical venous return across the foramen ovale via the ductus venosus, providing novel information regarding the spiraling course of flow through the ductus venosus and confirming the mechanism that results in a gradient in oxygen saturations between the right and left heart. Darby et al. (51) then used 4D flow in combination with T2 oximetry to show that pharmacologically induced dilation of the ductus venosus results in an increased shunting of umbilical venous return through the ductus venosus without resulting in increased cerebral oxygen delivery. This method was also applied to visualize hepatic blood flow (152) and changes in flow through the ductus arteriosus at birth (49). Owing to motion corruption during human fetal MRI, direct 3D imaging is often challenging. To address this issue, slice-to-volume reconstruction (SVR) combines multiple stacks of slices with co-registration allowing for volumetric fetal imaging. Multi-slice multiplanar accelerated PC MRI via golden-angle radial acquisition allows retrospective real-time image reconstruction with a high temporal and spatial resolution enabling motion correction and fetal heart rate estimation (32, 159, 160). Flow-sensitive cine imaging can be reconstructed and combined into a 4D flow volume with SVR, allowing comparable visualization of human fetal streaming patterns (33), as shown in Figure 6.

#### Ventricular volumetry

**Figure 7:**
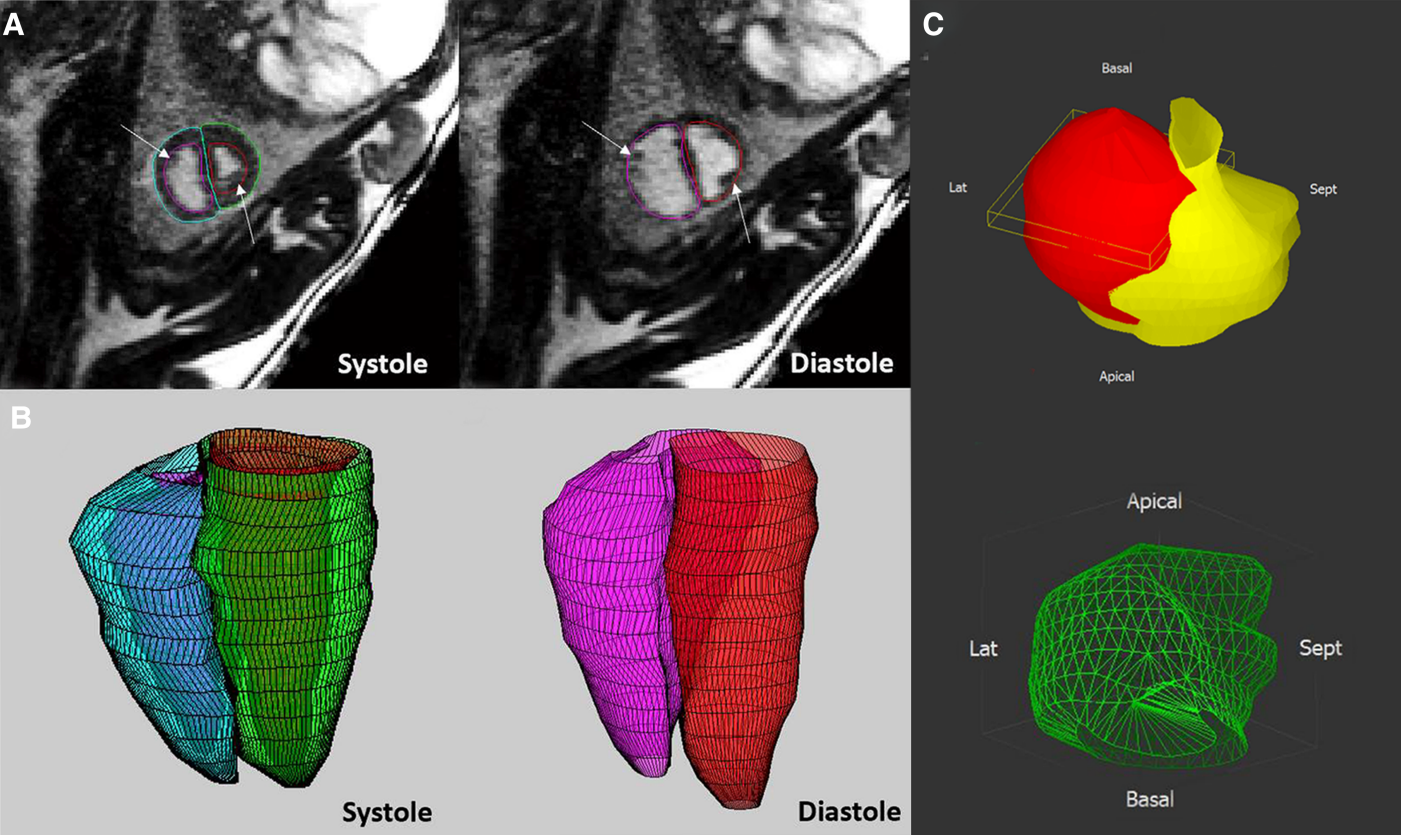
The application of ventricular volumetry in late gestation fetal sheep (**A,B**) and human fetus (**C**). (**A,B**) In fetal sheep, manual endocardial contours were applied on the stack of short-axis cine acquisitions in systole and diastole (**A**) to generate a 3D reconstruction of the right and left ventricles (**B**). (**C**) Morphological and quantitative models (green, mass 40 mm^3^) of a human fetal heart with a cardiac rhabdomyoma (red), and the compressed left ventricle (yellow). Adapted from (31) (**A,B**) and (40) (**C**).

A key component of the assessment of ventricular function by CMR is ventricular volumetry, whereby the endocardial and epicardial borders of the ventricles are contoured throughout a stack of cine MR images to measure right and left ventricular myocardial mass, end-systolic, end-diastolic and therefore stroke volumes as well as ejection fractions and CVO (31, 161). In late gestation anesthetized fetal sheep, ventricular volumetry can be obtained using a standard balanced steady state free precession (bSSFP) sequence with contiguous short axis cine images acquired using the blood pressure waveform obtained from an arterial catheter for cardiac triggering. This approach has confirmed the larger end-diastolic volume and stroke volume of the right ventricle than the left in the fetal circulation. Of note, right and left ventricular ejection fractions are not significantly different in the prenatal heart, presumably due to differences in loading conditions. The application of ventricular volumetry in human fetuses has been described in case reports as shown in Figure 7. However, this approach has not yet been reported in any systematic way due to the challenges to accurate ventricular volumetry posed by fetal motion (40).

#### Myocardial strain

**Figure 8:**
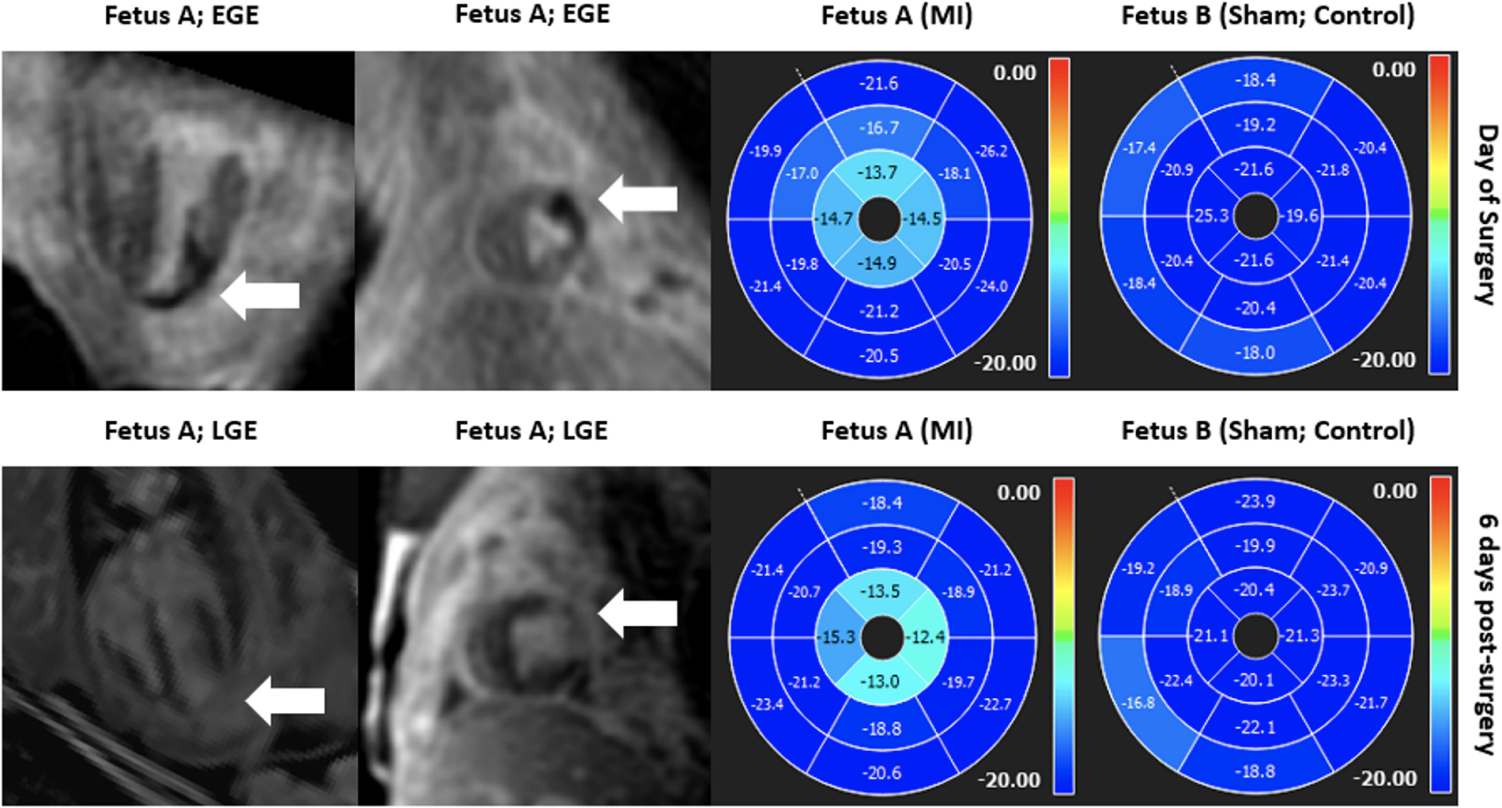
Gadolinium imaging (left) and SSFP short-axis cine feature tracking analysis (right) in a fetal sheep model of myocardial infarction on the day of surgery and 6 days post-surgery in both injured and sham twins suggesting good correlation between regional myocardial dysfunction in the apical left ventricular lateral wall and evidence of myocardial infarction in the respective segments in early and late gadolinium enhancement imaging. Anterolateral ischemia was noted at day of surgery from apex to near-mid ventricle (top row; early gadolinium enhancement; white arrow) and there was regional myocardial dysfunction in apical segments as measured by decreased circumferential strain by feature tracking (top row; right). 6 days after the myocardial infarction surgery, injury was noted in similar regions (bottom row; late gadolinium enhancement; white arrow) and there was regional myocardial dysfunction in the same regions, overall showing good correlation between injury and regional dysfunction measured by regional circumferential strain by feature tracking. In both scans, the internal sham control (fetus B) remained asymptomatic and did not demonstrate any regional wall motion abnormalities.

CMR also offers ways of measuring myocardial strain and can be broadly categorized into myocardial tagging and feature tracking. Myocardial tagging works by creating locally induced perturbations of the magnetization of the myocardial tissue prior to image acquisitions and these intrinsic markers, known as tags, move with the underlying tissue allowing quantification of myocardial deformation (162, 163). Tagging is widely accepted as the reference standard in the CMR community and has been validated extensively (164–172). However, it requires a dedicated sequence and time-consuming prost-processing. Moreover, the tags are typically deposited at detection of the QRS complex and introduces approximately a 30 ms delay, which may be particularly limiting in fetal population that has a rapid heart rate, underestimating myocardial strain (163). On the other hand, feature tracking does not necessitate a dedicated sequence and can be readily applied on typically acquired SSFP cine acquisitions (173, 174). Since its introduction in 2009, feature tracking has gained wide acceptance and has been extensively validated against myocardial tagging (175–179). However, given the current lack of standardization of methods and softwares for data analysis, its clinical translation is sparse and is mostly limited to research-oriented environment. CMR myocardial strain may prove useful in assessing regional myocardial dysfunction, and feature tracking has been explored as an approach to detecting wall motion abnormalities in a fetal sheep model of myocardial infarction. In our model, twin sheep fetuses were included in the study, whereby a branch of the left anterior descending coronary artery was ligated to induce a myocardial infarction with the other fetus serving as a sham control (44), as seen in Figure 8.

## Limitation

The accurate assessment of fetal heart function can be challenged by technical factors arising from the small size of the fetal heart, frequent fetal body motion and high fetal heart rate. The reliability of post-processing techniques for assessing cardiac function depends on image quality and structural detail, which has limited the routine application of techniques like speckle tracking for the clinical assessment of fetal cardiac function. Similar factors have limited the widespread adoption of fetal CMR. Compared with ultrasound, MRI is more expensive and less portable, and a clinical role for fetal CMR has not yet been established. MRI is typically more time consuming to acquire and process than ultrasound, which is exacerbated in the setting of fetal imaging due to artifacts resulting from fetal motion (18). A major limitation of fetal CMR arises from the intrinsic tradeoff between obtaining an adequate SNR to resolve small structures and high heart rates, while attempting to overcome artifacts arising from fetal motion by limiting scan time (34). In addition, organizational factors such as the availability of equipment and personnel to conduct fetal CMR must also be considered.

## Conclusions

Functional assessment of the fetal heart can be undertaken using a range of techniques. Conventional grey-scale ultrasound imaging is typically used to gain a subjective impression of global cardiac function, while 2D speckle tracking for strain imaging has provided a promising new approach to generating a quantitative assessment of ventricular function in a routine clinical setting. Fetal CMR represents an exciting development with the potential to augment the assessment of fetal cardiac function through techniques like ventricular volumetry and feature tracking. However, challenges to the widespread implementation of these approaches arise from the limitations imposed by fetal imaging, including the small size of the fetal heart, high heart rates and difficulties in obtaining standard views with adequate image quality. Further efforts to improve fetal cardiac imaging will be needed to exploit the full potential of fetal cardiac functional assessment, which is an important objective in the setting of advances in fetal cardiac diagnosis and therapy.

**Table 1:** Reference ranges for LV and RV longitudinal strain and strain rate by echocardiography in healthy fetuses reported in the literature.

Study	Parameters	*n*	GA (weeks)	Successful strain analysis (%)	RV average strain (%)	LV average strain (%)	RV average strain rate (1/s)	LV average strain rate (1/s)
Di Salvo et al. 2005 (141)		120	17–40	62.5	19 ± 8	17 ± 7	2.1 ± 0.8	2.1 ± 0.9
Di Salvo et al. 2008 (4)		100	20–32	100	−24 ± 4	−25 ± 4	NA	NA
Ta-Shma et al. 2008 (118)		28	20–38	94	21 ± 5	18.9 ± 5.7	2.3 ± 0.5	2.3 ± 0.7
Peng et al. 2009 (80)		151	18–40	87	NA	−17.78 ± 4.04	NA	−2.19 ± 0.65
Barker et al. 2009 (96)		33	17–38	100	−18.0 ± 6.4	−17.7 ± 6.4	−1.9 ± 0.8	−2.4 ± 1.2
Pu et al. 2010 (139)		170	20–41	89	−23.26 to −24.77	NA	−2.49 to −2.71	NA
Van Mieghem et al. 2010 (121)		55	16.9–36	83	−18.5 ± 6.8	−15.1 ± 5.2	−2.37 ± 0.93	−1.82 ± 0.68
Matsui et al. 2011 (78)	High FR (27.4–167.2 fps)	93	14–39	86	−22.3	−21.6	NA	NA
	Low FR (25 fps)			76	−23.2	−19.6	NA	NA
Willruth et al. 2011 (142)		150	13–39	98	−35.88 ± 11.21	−26.01 ± 6.38	−5.43 ± 2.41	−3.69 ± 2.41
Germanakis et al. 2012 (12)		144	14–39	83–85	−22.0 ± 3.7	−21.9 ± 3.7	NA	NA
Ishii et al. 2012 (138)		81	17–42	77–79	−16.0 ± 3.3	−15.2 ± 2.7	NA	NA
Kim et al. 2013 (143)		122	19–36	78	−22.6 ± 5.0	−21.5 ± 5.5	−2.6 ± 0.7	−2.5 ± 0.7
Kapusta et al. 2013 (127)	Longitudinal	78	20–24	96.2	−25.35 ± 4.03	−24.89 ± 4.57	−2.76 ± 0.62	−2.93 ± 0.88
		49	30–34	89.8	−23.20 ± 4.12	−24.68 ± 4.81	−2.29 ± 0.46	−2.58 ± 0.71
Maskatia et al. 2016 (144)		60	20–21	98.3	−18.82 ± 3.13	−19.61 ± 3.71	−2.04 ± 0.70	−2.15 ± 0.60
			24–25	93.3	−18.16 ± 2.95	−20.08 ± 2.66	−1.78 ± 0.41	−2.03 ± 0.43
			28–29	88.3	−19.47 ± 2.93	−20.95 ± 2.92	−1.78 ± 0.41	−2.00 ± 0.41
			32–33	86.7	−19.30 ± 2.75	−20.40 ± 3.13	−1.68 ± 0.37	−1.88 ± 0,37
			36–37	86.7	−19.54 ± 2.56	−21.13 ± 2.90	−1.68 ± 0.33	−1.98 ± 0.40
Chelliah et al. 2016 (117)	Longitudinal	58	12–14.5	36	−14.4 ± 5.5	−13.9 ± 5.7	NA	NA
		40	20–28	100	10	10	NA	NA
Dahlbäck et al. 2016 (145)		250	19–42	99.2	−14.6 ± 4.1	−15.1 ± 4.0	NA	NA
Enzensberger et al. 2017 (113)	High FR (60 fps)	117	17–39	86.3	−16.47	−17.06	NA	NA
	Low FR (30 fps)				−16.07	−17.54	NA	NA
Enzensberger et al. 2017 (146)		33	18.3–36.6	88	−14.65	−16.34	NA	NA
Li et al. 2017 (147)		102	15–40	73	NA	−22.3 ± 4.3	NA	−1.4 ± 0.5
DeVore et al. 2018 (148)		200	20–40	100	−22.70 ± 4.07	−22.93 ± 3.52	NA	NA
Alsolai et al. 2018 (111)	Longitudinal	276	36	89.8	−14.2 ± 3.4	−14.6 ± 3.8	−1.2 ± 0.2	−1.2 ± 0.3
			38	76.4	−13.4 ± 3.0	−13.6 ± 3.3	−1.1 ± 0.2	−1.1 ± 0.3
			40	85.1	−12.8 ± 2.8	−12.3 ± 3.1	−1.1 ± 0.2	−1.0 ± 0.3
Erickson et al. 2019 (149)	Longitudinal	50	16–20	90	−20,7	NA	−1.8	NA
			21–25		−18.3	NA	−1.5	NA
			26–29		−18.8	NA	−1.5	NA
			30–35		−18.4	NA	−1.4	NA
			36–40		−15.6	NA	−1.3	NA
Luo et al. 2021 (79)		59	21.6–36.6	100	−18.9 ± 1.5	−19.8 ± 1.5	NA	NA

NA, not available; RV, right ventricle; LV, left ventricle; FR, frame rate.
